# A clinical audit to investigate polypharmacy and interactions in inpatients in an old age psychiatric ward

**DOI:** 10.1192/bjo.2021.298

**Published:** 2021-06-18

**Authors:** Mohan Rathnaiah, Beth Brailsford, Nisha Mokashi

**Affiliations:** 1 Derbyshire Healthcare NHS FT; 2 UHDB NHS Founation Trust; 3 Nottinghamshire Healthcare NHS Foundation Trust

## Abstract

**Aims:**

To identify any problematic polypharmacy in the patient records of those staying in Cherry Ward, an old age psychiatric unit at Highbury Hospital, Nottingham in the calendar year 2018.

**Background:**

Multi-morbidity is defined as more than one long-term medical condition in a single individual and is a factor that is closely associated with polypharmacy, the use of multiple medications concurrently. Appropriate and Problematic are the two classifications of polypharmacy outlined by the King's Fund report, the first describing optimised evidenced-based pharmacological management of comorbidities and the latter to label prescribed medications whose use is not in the best interests of the patient. The risk of drug interactions and adverse drug reactions is increased with polypharmacy, and frail elderly patients are particularly at risk of the side-effects of psychotropic medications used in the management of mental health disorders. Guidelines highlight this group as a key party to be identified when searching for at-risk people.

**Method:**

The electronic records of those admitted and discharged from Cherry Ward in 2018 were reviewed in the period spanning January to May 2019, and the first forty-three patients were analysed in Microsoft Excel using criteria based on the King's fund report and the Medscape and BNF (British National Formulary) drug interaction tools. The Medscape drug interaction checker was used for initial screening; the complete medication list for each patient was entered into it and the number of interactions was displayed with advice on severity. If necessary, the individual interactions for each specific medication could also be cross-referenced in the BNF using the extensive lists provided for each drug. These are also graded from mild to severe.

**Result:**

On discharge, 69.7% (thirty patients) met the criteria for being at higher risk of polypharmacy. One patient became at higher risk of polypharmacy during admission, and another two stepped down from meeting the criteria on admission but not on discharge. Thirty-one of the forty-three patients had at least one interaction recorded; 18.6% (eight patients) had at least one potentially severe interaction.

**Conclusion:**

A substantial proportion of patients in Cherry ward in 2018 were at a higher risk of polypharmacy, reflecting current practice as outlined in the King's Fund report. Problematic polypharmacy is common among older patients hospitalised with psychiatric illness. Recommendations include use of an automated electronic system to investigate and flag up problematic polypharmacy and severe medication interactions.

